# In Vitro Fermentation Characteristics and Fiber-Degrading Enzyme Kinetics of Cellulose, Arabinoxylan, *β*-Glucan and Glucomannan by Pig Fecal Microbiota

**DOI:** 10.3390/microorganisms9051071

**Published:** 2021-05-16

**Authors:** Yu Bai, Xingjian Zhou, Na Li, Jinbiao Zhao, Hao Ye, Shiyi Zhang, Hongjian Yang, Yu Pi, Shiyu Tao, Dandan Han, Shuai Zhang, Junjun Wang

**Affiliations:** 1 State Key Laboratory of Animal Nutrition, College of Animal Science and Technology, China Agricultural University, Beijing 100193, China; yubaijlucau@163.com (Y.B.); zhouxingjiancd@163.com (X.Z.); swunln@163.com (N.L.); 15600911358@163.com (J.Z.); ye.hao5591@outlook.com (H.Y.); zhangshiyi2014@163.com (S.Z.); yang_hongjian@sina.com (H.Y.); yzupiyu@163.com (Y.P.); sytao@mail.hzau.edu.cn (S.T.); handandan@cau.edu.cn (D.H.); zhangshuai16@cau.edu.cn (S.Z.); 2 State Key Laboratory of Biological Feed, Ministry of Agriculture and Rural Affairs, Boen Biotechnology Co., Ltd., Guanzhou 341000, China

**Keywords:** non-digestible polysaccharides, microbiota, fermentation characteristic, fiber-degrading enzyme kinetics, short-chain fatty acids, pig

## Abstract

Non-digestible polysaccharides are of great significance to human and animal intestinal health. Cellulose, arabinoxylan, *β*−glucan and glucomannan were selected in the present study to investigate the fermentation characteristics and fiber-degrading enzyme kinetics by inoculating pig fecal microbiota in vitro. Our results showed that fermentation of arabinoxylan and *β*-glucan produced the highest amount of acetate and lactate, respectively. The abundance of *Prevotella_9* was the highest in *β*-glucan group and positively correlated with lactate and acetate. Glucomannan fermentation produced the highest amount of butyrate, and the abundance of *Lachnospiraceae_XPB_1014_group* and *Bacteroides* were the lowest. A significant negative correlation was found between *Lachnospiraceae_XPB_1014_group*, *Bacteroides* and butyrate. Exo*-β*-1,4-xylanase had the highest activity at 24 h during arabinoxylan fermentation. The activity of *β*-glucosidase and *β*-mannosidase at 36 h were higher than those at 15 h in the glucomannan group. The abundance of *Prevotella_9* was positively correlated with *β*-glucosidase while *Lachnospiraceae_XPB_1014_group* and *Bacteroides* were negatively correlated with *β*-xylosidase. Our findings demonstrated the *β*-glucan and arabinoxylan promote proliferation of *Prevotella_9*, with the preference to secret *β*-glucosidase, *β*-mannosidase and the potential to produce lactate and acetate. Butyrate production can be improved by inhibiting the proliferation of *Lachnospiraceae_XPB_1014_group* and *Bacteroides*, which have the lack of potential to secret *β*-xylosidase.

## 1. Introduction

Non-digestible polysaccharides cannot be digested in the small intestine but can be fermented by microbes in the gastrointestinal tract (GIT). Intake of non-digestible polysaccharides induces the changes in microbiota and metabolites in GIT, which in turn affects the nutritional, physiological and immunological functions of animals and humans [1,2]. The beneficial metabolites of non-digestible polysaccharide fermentation were mainly lactate and short-chain fatty acids (SCFA), including acetate, propionate and butyrate [3]. Acetate and propionate are involved in the energy metabolism of the host and serve as substrates for lipogenesis and gluconeogenesis in the liver and peripheral organs. Butyrate is not only helpful for reducing inflammatory bowel disease incidence but also an energy source for colonic mucosa cells [4].

Cellulose, arabinoxylan, *β*-glucan and glucomannan are important and typical non-digestible polysaccharides, which are the main ingredients of pig diets. Cellulose has been proved to have the ability to prevent gut inflammation and protect against dextran sodium sulfate-induced colitis [5,6]. Arabinoxylan plays an important role in maintaining the integrity of the gut by increasing the proliferation of goblet cells as well as secretion of IgA. *β*-glucan not only could improve metabolic condition, but also could attenuate cognitive impairment [7,8,9]. Glucomannan is helpful for bone metabolism and immunity [10]. The beneficial effects of non-digestible polysaccharides are closely related to their fermentation characteristics, including metabolites production and microbial regulation.

Recently, more and more attention had been paid to the fermentation characteristic of non-digestible polysaccharides; the gas and SCFA production ability of 12 non-digestible polysaccharides during fermentation were investigated, and found the high gas and SCFA production ability of cellulose [11]. However, low gas and SCFA production during cellulose fermentation was also found in previous research [12]. The fermentation characteristic of cellulose needs to be further investigated, and both research articles neglected the microbial regulation ability of cellulose. In addition, there is inconsistent research into the fermentation characteristic of arabinoxylan, *β*-glucan and glucomannan; the result of microbial regulation was also absent. Microbes in the GIT secrete various fiber-degrading enzymes to degrade non-digestible polysaccharides into monosaccharides, which are then converted into SCFA. The supplementation of fiber-degrading enzymes is beneficial to non-digestible polysaccharide degradation and SCFA production [13]. The role of fiber-degrading enzymes cannot be neglected during the fermentation of non-digestible polysaccharides. The fiber-degrading enzyme kinetics of cellulose, arabinoxylan, *β*-glucan and glucomannan during fermentation had never be published in previous studies [11,12,14].

The purpose of this research was to improve feed efficiency and the intestinal health of pigs through investigating the fermentation characteristics and fiber-degrading enzyme kinetics of cellulose, arabinoxylan, *β*-glucan and glucomannan by in vitro fermentation method. Gas production, pH changes and SCFA production were investigated in this research. In addition, microbial regulation, the activity of fiber-degrading enzymes, the correlations between metabolites and fiber-degrading enzymes with microbes were also included.

## 2. Materials and Methods

### 2.1. Substrates

Commercial grade non-digestible polysaccharides were used in this research. Cellulose, arabinoxylan, *β*-glucan and glucomannan were purchased from the Pioneer biotech company (Xi’an, China); they are originally from wheat, corn, oat and konjac (Batch Number: PB20190102, PB20180921, ZDY190308, HDFB15404), respectively. The purity of these non-digestible polysaccharides was 98%, 95%, 95% and 93% individually.

### 2.2. Preparation of Inocula

The process of inocula preparation was based on previous research [15]. Briefly, eight healthy pigs (Duroc × Landrace × Large White, 4 males and 4 females, bodyweight: 30 kg approximately) were selected to serve as the source of fecal inoculum. Animals were fed with a standard corn-soybean meal and without the use of antibiotics in the last three months before feces collection. Feces were collected directly from the rectum of pigs and immediately stored in plastic containers pre-filled with CO_2_. An equal amount of feces was taken from each animal, and then mixed and diluted six times (*m*/*v*) with pre-warmed (39 °C) sterile anaerobic saline (9 g/L NaCl). The diluted mixture was stirred for 1 min using a homogenizer and filtered through the four layers of sterile gauze which served as inocula. All the processes were finished within 1 h after feces collection and operated in an anaerobic operator (DWS, Whitley, England) which was filled with CO_2_ at 39 °C. The inocula were added into 10% glycerine (*v*/*v*) and separated into two parts, then stored at −80 °C after rapidly freezing with liquid nitrogen until further cumulative gas production trial and in vitro fermentation trial [16].

### 2.3. Cumulative Gas Production Trial

Cellulose, arabinoxylan, *β*-glucan and glucomannan were weighed at 0.5 g and served as substrates for fermentation. The medium for the cumulative gas production trial was prepared based on the previous study (the buffer solution was formulated with 8.32 g/L NaHCO_3_, 0.95 g/L NH_4_HCO_3_, 1.36 g/L Na_2_HPO_4_, 1.47 g/L KH_2_PO_4_, 0.14 g/L MgSO_4_·7H_2_O, 0.30 g/L Na_2_S·9H_2_O, 76.09 mg/L NaOH, 15.69 mg/L CaCl_2_·2H_2_O, 11.89 mg/L MnCl_2_·4H_2_O, 1.19 mg/L CoCl_2_·6H_2_O, 9.51 mg/L FeCl_3_·6H_2_O and 1.19 mg/L resazurin) [17]. Substrates were weighted in 100 mL serum bottles exactly, blended with 82 mL sterile medium, and then 5 mL melted inoculum was inoculated into a 100 mL serum bottle. Serum bottles without substrate were used as the blank control (*n* = 5). All steps were operated in the same condition as the inoculum producing process. The bottles were introduced with anaerobic N_2_ for 5 s, sealed, and incubated at 39 °C for 56 h in an air-ventilating temperature-controlled incubator. Each bottle was connected to a gas channel inlet of an automated gas production recording system designed by the College of Animal Science and the Technology of China Agricultural University. A 3.0 mL calibrated cumulative gas production for each vent was automatically recorded with a differential pressure switch (pressure range: 20–300 Pa, Huba Control Inc., Zurich, Switzerland) when the pressure inside the bottle increased up to 100 Pa [18].

Gas production profiles were fitted to the model described by Groot et al. [19] as:

(1) GPt=A1+(Ct)B

(2) $$R_{max}G=ABC^{B}\frac{TR_{max}G^{-B-1}}{{\left[1+C^{B}\times TR_{max}G^{-B}\right]}^{2}}$$

(3) TRmaxG=C[B−1B+1]1B

wher*e GP_t_* (mL/g) is the total gas produced per gram of sample, *A* (mL/g) is the asymptotic gas production per gram of sample, *B* is the switching characteristic of the curve, *C* is the time at which half of the asymptote has been reached (T_1/2_), and *t* is the time (h). *RmaxG* (mL/h) is the maximum rate of gas production. *TRmaxG* (h) is the time at which this maximum rate is reached.

### 2.4. In Vitro Fermentation Trial

The substrates, medium, inoculum and temperature were the same as those used in the cumulative gas production trial. Samples were collected at 0 h (beginning of fermentation), 15 h (prior stage of fermentation), 24 h (middle stage of fermentation) and 36 h (latter stage of fermentation) based on the result of the gas production trial (n = 5). Twenty milliliters of fermentation broth was taken from a serum bottle for pH detection (METTLER TOLEDO, Zurich, Switzerland) at sampling time, then was rapidly frozen with liquid nitrogen and stored at −80 °C for further analysis.

### 2.5. Samples Detection

#### 2.5.1. Monosaccharides Composition

Monosaccharide composition of cellulose, arabinoxylan, *β*-glucan and glucomannan including glucose, galactose, mannose, arabinose and xylose were measured according to the previous method [20]. Briefly, the biomass of four polysaccharides was extracted with water and ethanol at 100 °C, then about 300 mg of dried biomass was weighted in a 100 mL Pyrex glass bottle. Throughout the process of the hydrolysis reaction, 72% sulfuric acid was incubated, and after 60 min deionized water was added. The mixture was autoclaved. Meanwhile, a sugar recovery standard was prepared. Released monosaccharides were analyzed using an ICS-3000 HPLC system (Thermo Fisher Scientific, Sunnyvale, CA, USA) equipped with a pulsed-amperometric detector. Samples were injected onto a 150 mm × 3 mm CarboPac PA20 column (Thermo Fisher Scientific, Sunnyvale, CA, USA) with a 50 mm × 3 mm guard column of the same material. Elution was performed at 30 °C with 2 mM potassium hydroxide at a flow rate of 0.4 mL/min.

#### 2.5.2. SCFA and Lactate Detection

The concentration of SCFA and lactate in the fermentation broth was analyzed following the previous method, and with slight modifications [21]. Briefly, the fermentation broth was diluted with ultrapure water, filtered using a 0.20 mm Nylon Membrane Filter (Millipore, Bedford, OH, USA), and then poured into a Gas Chromatograph System (Agilent HP 6890 Series, Santa Clara, CA, USA). Lactate was quantified by HPLC. An Ultimate 3000 HPLC (Dionex) is equipped with a RI-101 refractive index detector (Shodex, Kawasaki, Japan), an autosampler and an ion-exclusion Aminex HPX-87H column (7.8 × 300 mm) with a guard column (Bio-Rad, Hercules, CA, USA). SCFA and lactate production at different sampling times were corrected by the concentration of SCFA and lactate in the fermentation broth at 0 h.

#### 2.5.3. Bacterial Community

Three samples were detected for the bacterial community at each time point because of the little difference in the production of SCFA within the group. The total microbial genomic DNA in the fermentation broth was extracted using the QIAamp Fast DNA Stool Mini Kit (Qiagen Ltd., Düsseldorf, Germany) following the manufacturer’s instructions. The detection method for the fermentation broth bacterial community was based on previous research [22]. Amplicon libraries were sequenced on the Illumina HiSeq 2500 platform (Illumina, San Diego, CA, USA) for paired-end reads of 250 bp. PANDAseq (version 2.9) was used to remove low-quality sequences [23]. The high-quality sequences were clustered into OTUs with a 97% similarity using UPARSE (version 7.0) [24] in QIIME (version 1.8) [25], and the chimeric sequences were removed by UCHIME [24]. Taxonomy was assigned to OTUs using the RDP classfier^1^ [26] against the SILVA 16S rRNA gene database (Release128^2^) [27] with a confidence threshold of 70%. Alpha-diversity was evaluated by calculating the Shannon, Chao diversity index and number of OTUs per sample with the MOTHUR program (version 1.30.1) [28]. The microbial sequencing data had been uploaded to the NCBI, and the BioProject ID is PRJNA687109.

#### 2.5.4. Determination of Fiber-Degrading Enzymes Activity

The detection kit of cellulase (ml076772), endo-*β*-1,4-glucanase (ml077334), *β*-glucosidase (ml076766), exo-*β*-1,4-xylanase (ml076752), α-L-arabinofuranosidases (ml076760) and *β*-xylosidase (ml077336) were used by the manufacturer’s instruction to detect the activity of fiber-degrading enzyme activity based on spectrophotometry (Shanghai Enzyme-linked Biotechnology, China). The activity of *β*-mannosidase (ml027495) was detected by an enzyme-linked immunosorbent assay kit (Shanghai Enzyme-linked Biotechnology, China).

### 2.6. Statistical Analysis

The data were analyzed using the SPSS software package (SPSS v. 20.0, SPSS Inc., Chicago, IL, USA). Microbial differences among groups were determined using the Kruskal–Wallis ANOVA test. Metabolites and enzyme differences among groups were determined using one-way ANOVA with Tukey’s post-hoc test. Statistical variations were estimated by the standard error of the means. The correlation coefficient between microbes and bacterial metabolites, as well as microbes and activities of fiber-degrading enzymes were analyzed by Spearman’s correlation. PCoA was performed based on Bray–Curtis distance using QIIME (version 1.8). ANOSIM based on Bray–Curtis distance was performed to compare the similarity of microbial community between groups using the “vegan” package of R (version 3.3.1). All statistical analyses were considered significant at *p* < 0.05.

## 3. Results

### 3.1. Monosaccharides Composition of Cellulose, Arabinoxylan, β-Glucan and Glucomannan

The monosaccharide composition of cellulose, arabinoxylan, *β*-glucan and glucomannan is presented in Table 1, which is generally in line with our expectations. The glucose content of cellulose was 96.62%, while xylose was determined as 3.29%. Arabinoxylan contained 33.42% arabinose and 65.38% xylose, with a 0.51 arabinose to xylose ratio. *β*-glucan used in this research only contained glucose, of which the content is 92.07%. Glucomannan consisted of four kinds of monosaccharides: 58.29% glucose, 28.82% mannose, 6.34% galactose and 2.59% xylose.

### 3.2. Gas Production and pH Shifts during Cellulose, Arabinoxylan, β-Glucan and Glucomannan Fermentation

**Figure 1:**
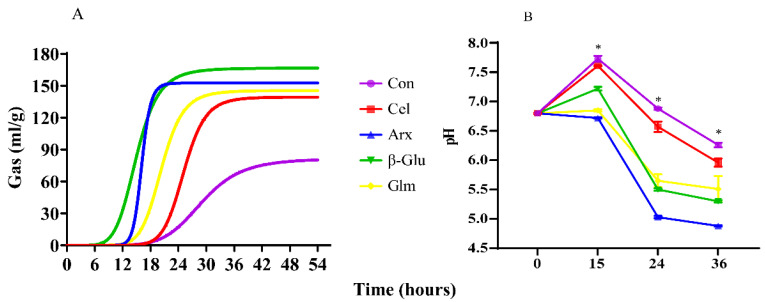
In vitro gas production curve (**A**) and pH changes (**B**) over time during fermentation. The results were analyzed by the one-way ANOVA with Tukey’s post hoc test (*n* = 5, * mean *p* < 0.05; Con, control; Cel, cellulose; Arx, arabinoxylan; *β*-Glu, *β*-glucan; Glu, glucomannan).

Gas production and pH shifts were monitored in this research (Figure 1). The gas production curve is shown in Figure 1A. Cellulose fermentation had the lowest asymptotic gas production and the longest half time of asymptotic gas production among four polysaccharides (*p* < 0.05) (Table 2). In addition, the pH of cellulose fermentation at 15 h, 24 h and 36 h were higher than the other polysaccharides (*p* < 0.05). The pH of the arabinoxylan group at 24 h and 36 h were the lowest among all polysaccharides (Figure 1B).

### 3.3. The Changes in Microbial Diversity during Cellulose, Arabinoxylan, β-Glucan and Glucomannan Fermentation

**Figure 2:**
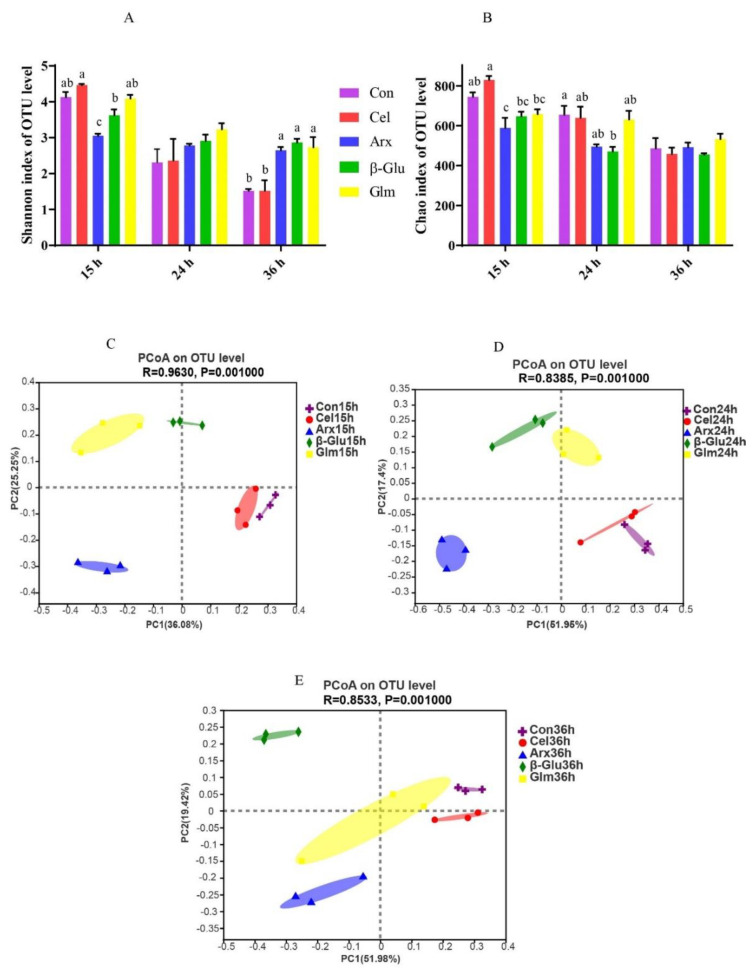
Effects of four polysaccharides on bacterial α-diversity (Shannon index, **A**; Chao index, **B**) and *β*-diversity (PCoA, 15 h, **C**; 24 h, **D**; 36 h, **E**) in the fermentation broth. The results were analyzed by the Kruskal–Wallis ANOVA test and presented as mean values; PCoA plot based on the Bray–Curtis distance matrix (*n* = 3; Different letters mean *p* < 0.05; Con, control; Cel, cellulose; Arx, arabinoxylan; *β*-Glu, *β*-glucan; Glu, glucomannan).

Alpha-diversity of microbiota in four polysaccharides’ fermentation was presented by Shannon (Figure 2A) and Chao indexes (Figure 2B). The Shannon index of cellulose was lower than that of arabinoxylan, *β*-glucan and glucomannan at 36 h during fermentation (*p* < 0.05) (Figure 2A). The principal coordinate analysis (PCoA) plots based on Bray–Curtis distance showed clear segregations on the microbiota structures among different groups at15 h, 24 h and 36 h (Figure 2C–E).

### 3.4. The Changes in the Microbial Composition during Cellulose, Arabinoxylan, β-Glucan and Glucomannan Fermentation

**Figure 3:**
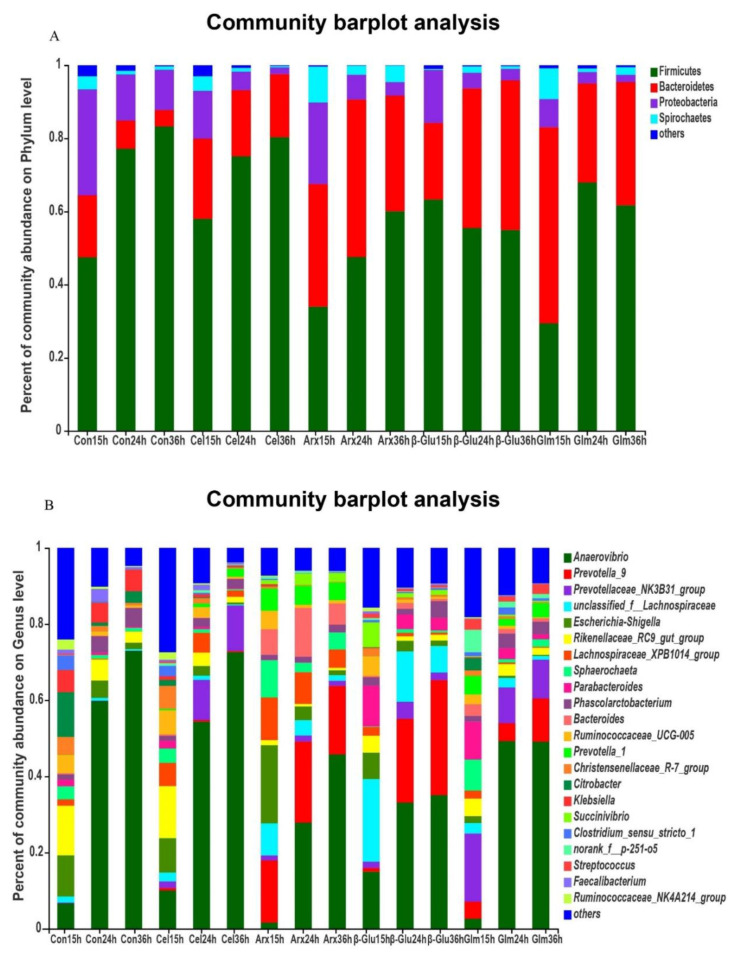
The microbial composition at the phylum and genus level in fermentation broth at 15, 24 and 36 h. The microbial community at the phylum level with an abundance greater than 0.01% in fermentation broth (**A**). The microbial composition at the genus level with an abundance greater than 1% in fermentation broth (**B**). (*n* = 3; Con, control; Cel, cellulose; Arx, arabinoxylan; *β*-Glu, *β*-glucan; Glu, glucomannan).

At the phylum level, the Firmicutes, Bacteroidetes and Proteobacteria were dominant bacteria in all groups (Figure 3A). However, the relative abundance of Proteobacteria was decreased along with the process of fermentation (Figure 3A). At the genus level, *Anaerovibrio* was the main bacteria in all groups (Figure 3B).

### 3.5. The Production of Lactate, SCFA and Their Relationship with the First Six Differential Bacteria

**Figure 4:**
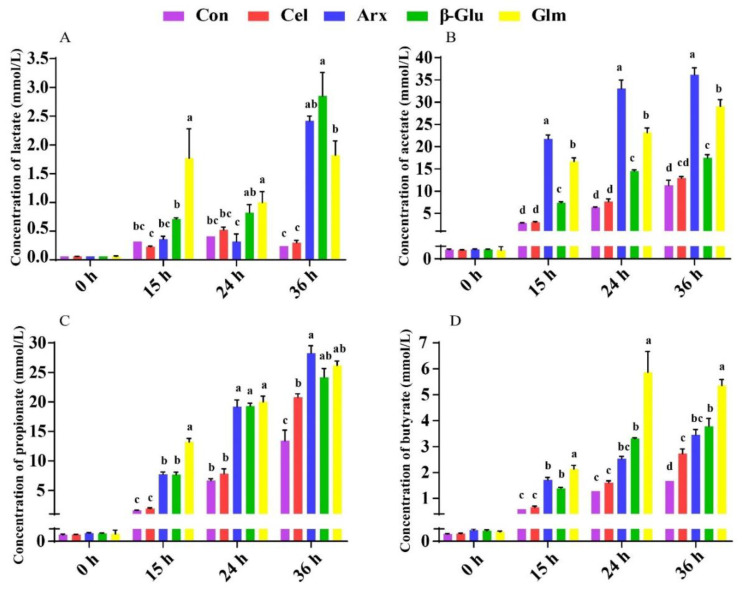
Quantification of lactate (**A**), acetate (**B**), propionate (**C**), and butyrate (**D**) in the fermentation broth of five groups at 0, 15, 24, 36 h during fermentation. All data were analyzed by the one-way ANOVA with Tukey’s post-hoc test and presented as the mean values (*n* = 5; Different letters mean *p* < 0.05; Con, control; Cel, cellulose; Arx, arabinoxylan; *β*-Glu, *β*-glucan; Glu, glucomannan).

**Figure 5:**
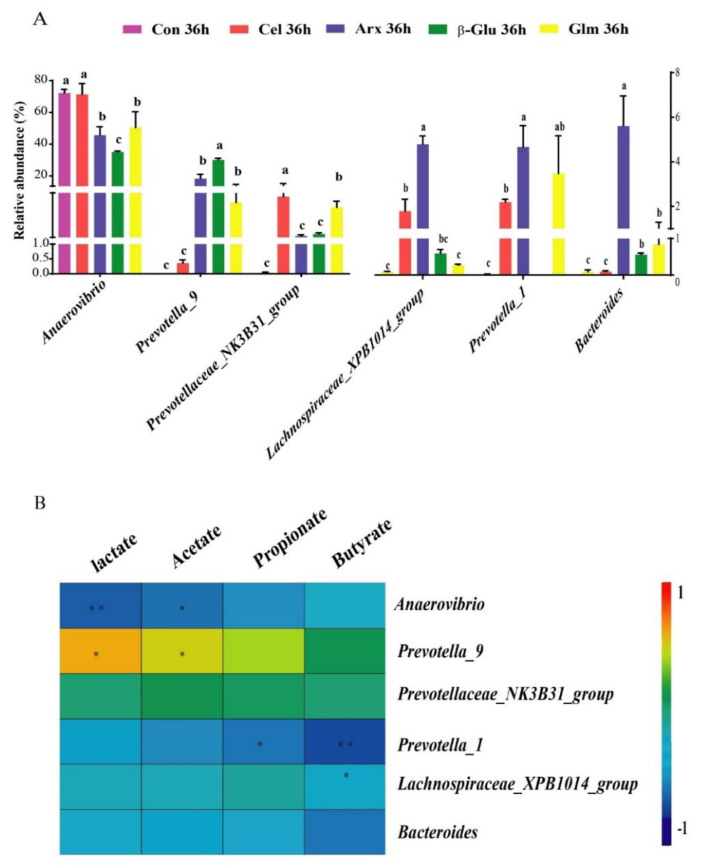
Analysis of the first six differential bacteria among five groups at the genus level and 36 h during fermentation (**A**) (n = 3). The spearman correlation between lactate, SCFA and the first six differential bacteria at 36 h (**B**). All data were analyzed by Kruskal–Wallis ANOVA test and presented as the mean values (*n* = 15; Different letters and * mean *p* < 0.05; ** means *p* < 0.01; Con, control; Cel, cellulose; Arx, arabinoxylan; *β*-Glu, *β*-glucan; Glu, glucomannan).

The amount of lactate and SCFA production was determined at four time points (Figure 4). We found *β*-glucan fermentation produced the highest amount of lactate among all polysaccharides at 36 h (*p* < 0.05) (Figure 4A). Furthermore, arabinoxylan fermentation produced more acetate than the other polysaccharides (*p* < 0.05) (Figure 4B), and glucomannan fermentation showed the highest ability to produce butyrate (*p* < 0.05) (Figure 4D). In addition, the microbial composition was impacted by different substrates, and we selected six genera that were most different among all groups at 36 h (*p* < 0.05, abundance > 0.5%) (Figure 5A). Spearman’s correlation analysis was applied between these six genera with the amount of lactate and SCFA (Figure 5B). The abundance of *Anaerovibrio* in *β*-glucan and arabinoxylan groups was lower than that in the cellulose group, but *β*-glucan and arabinoxylan groups contained more *Provetella_9* than the cellulose group (*p* < 0.05) (Figure 5A). The relative abundance of *Lachnospiraceae_XPB1014_group* and *Bacteroides* in the arabinoxylan group were higher than those in the other polysaccharides (*p* < 0.05) (Figure 5A). Results of a correlation analysis showed a negative correlation between *Anaerovibrio* with lactate (*p* < 0.01) and acetate (*p* < 0.05). However, lactate and acetate were positively correlated with *Provetella_9* (*p* < 0.05) (Figure 5B). Furthermore, *Lachnospiraceae_XPB1014_group* (*p* < 0.01) and *Bacteroides* (*p* < 0.05) were negatively correlated with butyrate (Figure 5B).

### 3.6. The Activity of Fiber-Degrading Enzymes and Their Relationship with Dominant Bacteria during Fermentation

**Figure 6:**
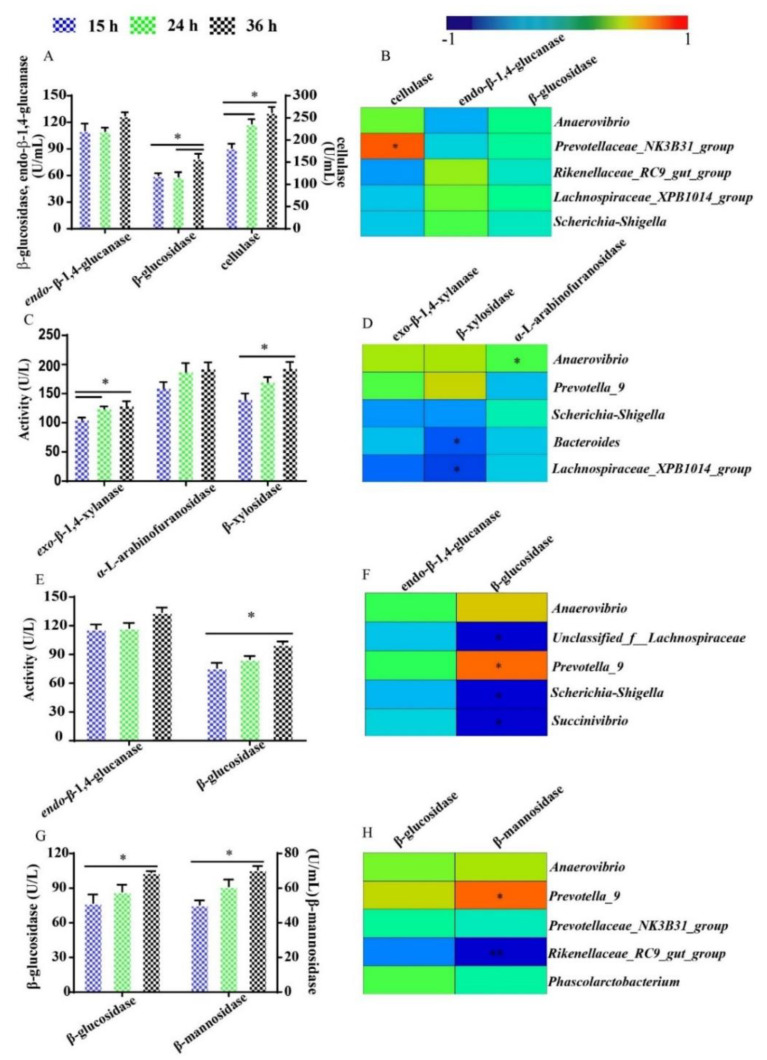
The activities of endo-*β*-1,4-glucanase, cellulase, and *β*-glucosidase (**A**), Spearman’s correlation between these enzymes and the top five bacteria at 15, 24 and 36 h during cellulose fermentation (**B**). The activity of exo-*β*-xylanase, *α*-L-arabinofuranosidases and *β*-xylosidase (**C**), Spearman’s correlation between these enzymes and the top five bacteria at 15, 24 and 36 h during arabinoxylan fermentation (**D**). The activity of endo-*β*-1,4-glucanase and *β*-glucosidase (**E**), Spearman’s correlation between these enzymes and the top five bacteria at 15, 24 and 36 h during *β*-glucan fermentation (**F**). The activity of *β*-glucosidase and *β*-mannosidase (**G**), Spearman’s correlation between these enzymes and the top five bacteria at 15, 24 and 36 h during glucomannan fermentation (**H**). All data were analyzed by the one-way ANOVA with Tukey’s post-hoc test and presented as the mean value (*n* = 9, * mean *p* < 0.05; ** means *p* < 0.01; Con, control; Cel, cellulose; Arx, arabinoxylan; *β*-Glu, *β*-glucan; Glu, glucomannan).

The activities of fiber-degrading enzymes during cellulose, arabinoxylan, *β*-glucan and glucomannan fermentation were monitored in this research, and Spearman’s correlation was processed between the fiber-degrading enzyme activity and the most abundant five bacteria with relative abundance over 5% throughout the fermentation process (Figure 6). Cellulase degrades cellulose into cellobiose which is then converted into glucose by *β*-glucosidase. There was no significant difference between the activity of cellulase at 36 h and 24 h, but the activity of *β*-glucosidase at 36 h was higher than that at 24 h in the cellulose group (*p* < 0.05) (Figure 6A). Furthermore, a significant positive correlation was found between cellulase and *Prevotellaceae_NK3B31_group* (*p* < 0.05) (Figure 6B). The activity of exo-*β*-1,4-xylanase at 24 h was higher than that at 15 h (*p* < 0.05), but no significant difference was found between the activity of it at 24 h and 36 h during arabinoxylan fermentation (Figure 6C). The activity of *β*-xylosidase at 36 h was higher than that at 15 h (*p* < 0.05), but the activity of *α*-L-arabinofuranosidases was not significantly different among all time points in the arabinoxylan group (Figure 6C). Moreover, *Bacteroides* and *Lachnospiraceae XPB_1014_group* were negatively correlated with *β*-xylosidase during arabinoxylan fermentation (*p* < 0.05) (Figure 6D). The activity of *β*-glucosidase at 36 h was higher than that at 15 h in the *β*-glucan group (*p* < 0.05) (Figure 6E). The activity of *β*-glucosidase presented a positive correlation with *Prevotella_9*, but a negative correlation with *unclassified_f__Lachnospiraceae*, *Escherichia-Shigella* and *Succinivibrio* in this research (*p* < 0.05) (Figure 6F). The activities of *β*-glucosidase and *β*-mannosidase at 36 h were higher than those at 15 h in the glucomannan group (*p* < 0.05) (Figure 6G). Besides, *β*-mannosidase was positively correlated with *Prevotella_9* (*p* < 0.05), but highly negatively correlated with *Rikenellaceae_RC9_gut_group* (*p* < 0.01) (Figure 6H).

## 4. Discussion

Diet induces changes in microbial ecology and fermentation end-products in the gut, which in turn affects the nutritional, physiological and immunological functions of the host [29]. Non-digestible polysaccharides are a major part of cereal grains and vegetables, which are ingested by humans and pigs, and are beneficial to intestinal health. Cellulose, arabinoxylan, *β*-glucan and glucomannan are the main non-digestible polysaccharides in the human and pig diet. A deep understanding of fermentation characteristics of non-digestible polysaccharides is helpful for feed utilization efficiently.

This research aimed to investigate the fermentation characteristics and fiber-degrading enzyme kinetics of cellulose, arabinoxylan, *β*-glucan and glucomannan by in vitro fermentation methods. Our result revealed that the fermentability of cellulose was the lowest, with the lowest gas production and the highest pH during fermentation. Arabinoxylan and *β*-glucans had the potential to produce acetate and lactate, respectively. Glucomannan promoted the production of butyrate by inhibiting the proliferation of *Lachnospiraceae_XPB_1014_group* and *Bacteroides*. Besides, the activity of fiber-degrading enzymes underwent dramatic changes at different time points during fermentation. We also observed the specific microbes were closely related to the production of metabolites and activity of fiber-degrading enzymes.

The monosaccharide composition of cellulose, arabinoxylan, *β*-glucan and glucomannan was investigated in this research. The extracted cellulose usually contains a small quantity of lignin, and xylose is the main monosaccharide of lignin [30]. Thus, the low content of xylose found in cellulose is assumed mainly from lignin. The source of arabinoxylan has a great impact on monosaccharide composition. The arabinose-to-xylose ratio in arabinoxylan from wheat and rye ranges from 0.50 to 0.70 and 0.48 to 0.55, respectively. However, rice (0.80) and sorghum (0.87) are heavily branched and contain more arabinose, galactose and glucuronic acid substituents [31]. Most of the *β*-glucans contain pure glucose, except for seaweed (laminarin) which also contains mannose. The presence of galactose and xylose may be attributable to galactomannan and xyloglucan mixed in glucomannan.

The shifts in gas production and pH are important indicators for the fermentability of non-digestible polysaccharides since the metabolic activity of microorganisms usually produce gas and metabolites including lactate and SCFA [32]. The cellulose group showed the lowest gas production among the four groups, indicating its lowest fermentability. The insolubility of cellulose might be the main reason for its low fermentability. Insoluble dietary fiber was considered to be fermented slower than soluble dietary fiber due to the low capacity of hydration [12]. Arabinoxylan, with high solubility, showed the best fermentability based on the lowest pH throughout the fermentation process.

Low fermentability could also be attributable to low microbial diversity indicated by the low Shannon index in cellulose fermentation process. Lower microbial diversity was also reported in cellulose fermentation compared with inulin fermentation, because of the lack of sufficient nutrients for microbes [33], such as the low amount of SCFA for microbes that can only remain alive by cross-feeding. Clear segregations on the microbiota structures among different groups might be caused by the selective polysaccharide’s utilization of bacteria during fermentation. The fermentation of non-digestible polysaccharides had a great impact on the microbiota at the phylum and genus level. The decreased level of Proteobacteria can be explained by the depolymerization of polysaccharides by microbiota since all polysaccharides could efficiently reduce the relative abundance of Proteobacteria [34]. *Anaerovibrio* was the dominant bacteria at the genus level, and was found to be significantly associated with feed efficiency [35]. The substrates used in this research are important components of feeds, and have a great influence on feed efficiency. *Anaerovibrio* could also produce lipase and lead to a reduction in lipolysis, thus lowering the amount of polyunsaturated fatty acids available for biohydrogenation [36].

The end products of non-digestible polysaccharide fermentation include lactate, SCFA, acetate, propionate, butyrate, and various gases like hydrogen, carbon dioxide and methane [29]. The lactate and SCFA are recognized as beneficial metabolites of polysaccharides fermentation. *β*-glucan has been proved to have the potential to produce lactate, which could not only increase the content of lactate in the mucus of piglets but also provoke the growth of *Bifidobacterium* and the emergence of a balanced microbiota [37]. Besides, *β*-glucan oligosaccharides showed great lactate-producing capacity [38]. The fermentation of arabinoxylan significantly promoted the production of acetate, which is consistent with the previous research because of its high solubility [39]. The low abundance of *Anaerovibrio,* but the high abundance of *Provetella_9,* might contribute to the mass production of lactate and acetate during *β*-glucan and arabinoxylan fermentation. The enrichment of *Anaerovibrio* followed by the decreased proportion of lactate and acetate was also found in in vitro fermentation trials [40,41]. Another ability of *Anaerovibrio* was to ferment glycerol into propionate and succinate, among which was propionate produced by the dicarboxylic acid pathway [42]. Positive correlations between lactate and acetate with *Provetella_9* were observed in the gut of multiple sclerosis patients [43]. *Provetella_9,* as a beneficial bacterium, was present in a low abundance in patients who had Clostridioides difficile infection [44]. The gut microbiome of multiple sclerosis patients was characterized by a decrease of *Prevotella_9*, with glatiramer acetate with acetate as the main ingredient that can prevent and suppress multiple sclerosis, suggesting that acetate may have an important relation with *Prevotella_9* [43,45]. Acetate, as the metabolite of *Prevotella_9,* played a vital role in preventing multiple sclerosis. Glucomannan and its degradation product oligo-glucomannan had been proved to increase the production of butyrate [46]. A negative correlation between *Bacteroides* and butyrate was found within in vitro trials [47]. The inhibition of *Lachnospiraceae_XPB1014_group* and *Bacteroides* were speculated as a reason why glucomannan fermentation has the potential to produce butyrate. In addition, the phenomenon of a high gene copy numbers of *Bacteroides,* but a low concentration of butyrate, was observed in the intestine of the pigs [48].

Fiber-degrading enzymes are secreted by bacteria and serve as significant “tools” to degrade polysaccharides into monosaccharides; then, monosaccharides were metabolized into SCFA by microbes. Fiber-degrading enzymes are specific, to degrade polysaccharides and have different changes of activity over time during fermentation. Cellulase can degrade cellulose into cellobiose which is then converted into glucose by *β*-glucosidase. This research proved that the preliminary degradation of cellulose may stop at 24 h, and cellobiose, the product of cellulose preliminary degradation, was degraded into glucose in the following process. We assumed that the activity of cellulase was inhibited by the accumulation of cellobiose, which showed no suppression of the activity of *β*-glucosidase, and then *β*-glucosidase was markedly accelerated by the hydrolyzation of cellobiose to glucose [49]. The significant positive correlation between cellulase and *Prevotellaceae_NK3B31_group* suggested that *Prevotellaceae_NK3B31_group* may have great cellulose degradation capacity, and may proliferate rapidly when stimulated by the presence of cellulose. In work with rats, the population of *Prevotellaceae_NK3B31_group* increased with the husks of Xanthoceras sorbifolia Bunge intake, and cellulose was the main non-digestible polysaccharides of Xanthoceras sorbifolia Bunge husks [50]. A higher level of *Prevotellaceae_NK3B31_group* in hindgut was also found in piglets fed with a high fiber diet rather than a low fiber diet [51], possibly because of the powerful cellulose degradation capacity of *Prevotellaceae_NK3B31_group*.

Exo-*β*-1,4-xylanase is essential in the first step of arabinoxylan degradation, and then *α*-L-arabinofuranosidases and *β*-xylosidase come into play before arabinose and xylose were metabolized into SCFA. The activity of exo-*β*-1,4-xylanase at 36 h was not higher than that at 24 h, indicating that the primary degradation of arabinoxylan mainly happened within the first 24 h of fermentation, which is in agreement with the founding of Barbara’s research that the activity of exo-*β*-1,4-xylanase also reached a peak at 24 h [52]. We could infer that the bacterial demand for xylose increased over time, while the requirement for arabinose was steady during the whole arabinoxylan fermentation process. The ratio of arabinose to xylose increased with the prolonging of arabinoxylan fermentation, which also indicated that xylose was easier to be metabolized by microbiota than arabinose [14]. *Bacteroides* and *Lachnospiraceae XPB_1014_group* were dominant bacteria during arabinoxylan fermentation, but *Bacteroides* and *Lachnospiraceae XPB_1014_group* had a negative correlation with *β*-xylosidase during arabinoxylan fermentation. However, the previous study found that the total amount of xylose in ileal digesta was positively correlated with the abundance of *Bacteroides* [53]. The possible reasons for the difference were synergistic metabolic and cross-feeding; synergistic metabolic and cross-feeding events have been widely proved in intestinal microecology, and the high amount of xylose in ileal digesta may be the product of degrading enzymes which are secreted by the other bacteria, except *Bacteroides* [54]. The relationship between *β*-xylosidase and *Lachnospiraceae XPB_1014_group* has never been reported. However, the abundance of *Lachnospiraceae_XPB1014_group* was negatively correlated with the bodyweight of pigs with a defect in fiber utilization, possibly because of the low xylose utilized capacity of *Lachnospiraceae_XPB1014_group* [55].

The increased activity of *β*-glucosidase showed that the metabolism of glucose for microbes was enhanced from 15 h to 36 h during *β*-glucan fermentation. The positive correlation between *Prevotella_9* and *β*-glucosidase was proved by a previous study, which found that *Prevotella_9* played an active role in glucose metabolism, and the changing trend of *Prevotella_9* was consistent with that of glucose [56,57]. *Escherichia-Shigella* has been known as harmful bacteria because of its poor ability to produce SCFA and could cause intestinal inflammation [58]. The negative correlation between *β*-glucosidase and *Escherichia-Shigella* has never been reported, but the proliferation of *Escherichia-Shigella* could be inhibited by traditional Chinese medicinal materials, which would then influenced glucose and amino acid metabolism [59].

*β*-glucosidase and *β*-mannosidase are the main enzymes for glucose and mannose degraded from glucomannan. The increased activities of *β*-glucosidase and *β*-mannosidase suggested that the demand for glucose and mannose in the glucomannan group increased significantly after 15 to 36 h of fermentation. The time of half of the asymptote reached was 20.39 h in the glucomannan group, indicating the high metabolic level activities of bacteria after 15 h of fermentation as well. The relationship between *Prevotella_9*, *Rikenellaceae_RC9_gut_group* and *β*-mannosidase indicated that mannose in the glucomannan group was more likely to be utilized by *Prevotella_9*, not *Rikenellaceae_RC9_gut_group*. Furthermore, the previous study showed *Prevotella_9* was rich in the hindgut of growing pigs, and associated with the increased enzymatic capacity for mannose metabolism [60]. The relationship between *Rikenellaceae_RC9_gut_group* and *β*-mannosidase has not been announced, but *Rikenellaceae_RC9_gut_group* was rich in the gut of rats that suffered from acute myocardial ischemia and impacted the intestinal permeability, oxidative stress, and energy metabolism [61].

This research investigated the relationships between microbial metabolites, fiber-degrading enzymes and microbes at the genus level. The ability of microbes to produce metabolites and secrete fiber-degrading enzymes needs to be clarified at the strain level in further research. The cooperative effect of microbes during the degradation of non-digestible polysaccharides is also worthy of study.

## 5. Conclusions

Cellulose showed the lowest fermentability, while arabinoxylan and *β*-glucan showed high fermentability and the potential to promote acetate and lactate production during fermentation by microbes from the hindgut of pigs. Arabinoxylan and *β*-glucan increased the relative abundance of *Prevotella_9*, which acted as acetate and lactate-producing bacteria and was found with the potential to secret *β*-glucosidase and *β*-mannosidase to degrade glucomannan during fermentation. The supplementation of glucomannan could increase the production of butyrate by inhibiting the proliferation of *Lachnospiraceae_XPB1014_group* and *Bacteroides*. The primary degradation of arabinoxylan happened within 24 h, and the *Lachnospiraceae_XPB1014_group* and *Bacteroides* in pigs’ hindguts showed a low *β*-xylosidase secretion capacity during the arabinoxylan fermentation.

## Figures and Tables

**Table 1:** Monosaccharide composition of cellulose, *β*-glucan, arabinoxylan and glucomannan (mol %).

Polysaccharides	Glucose	Galactose	Mannose	Arabinose	Xylose
cellulose	96.62	0.00	0.00	0.00	3.29
arabinoxylan	2.02	0.00	0.00	33.42	65.38
*β*-glucan	92.07	0.00	0.00	0.00	0.00
glucomannan	58.29	6.34	28.82	0.00	2.59

All data are the results of chemical analysis conducted in duplicate.

**Table 2:** The fermentation kinetics of different polysaccharides in cumulative gas production trial.

Items	Con	Cel	Arx	*β*-Glu	Glm	*p*-Value
A, mL/g	81.55 ± 8.95 ^c^	139.37 ± 3.74 ^b^	152.69 ± 3.89 ^a^	166.66 ± 3.71 ^a^	145.52 ± 3.74 ^a^	<0.01
B	6.80 ± 1.09 ^d^	10.96 ± 0.26 ^b^	16.84 ± 0.78 ^a^	6.31 ± 0.19 ^d^	8.85 ± 0.56b ^c^	<0.01
C, h	29.11 ± 0.36 ^a^	25.19 ± 0.46 ^b^	16.12 ± 0.15 ^d^	14.97 ± 0.27 ^d^	20.39 ± 0.38 ^c^	<0.01
RmaxG, mL/h	27.65 ± 0.26 ^a^	24.77 ± 0.44 ^b^	16.01 ± 0.14 ^d^	14.22 ± 0.26 ^e^	19.86 ± 0.32 ^c^	<0.01
TRmaxG, h	4.78 ± 0.61 ^c^	15.32 ± 0.71 ^b^	39.95 ± 1.79 ^a^	17.99 ± 0.24 ^b^	16.03 ± 1.29 ^b^	<0.01

The different alphabets in a row mean a significant difference (*p* < 0.05). *n* = 5. A (mL/g) is the asymptotic gas production per gram of sample. B is the switching characteristic of the curve. C is the time at which half of the asymptote has been reached (T_1/2_), and t is the time (h). RmaxG (mL/h), the maximum rate of gas production. TRmaxG (h), the time at which the maximum rate is reached. Con, control; Cel, cellulose; Arx, arabinoxylan; *β*-Glu, *β*-glucan; Glm, glucomannan.

## Data Availability

The microbial sequencing data has been uploaded to the NCBI, and the BioProject ID is PRJNA687109.
